# Yellow Nail Syndrome with Bilateral Pleural Plaques and Diffuse Pleural Thickening: A Mimic of Asbestos Related Disease

**DOI:** 10.1155/2018/7302898

**Published:** 2018-09-24

**Authors:** Adam Dallmann, Richard L. Attanoos

**Affiliations:** ^1^University Hospital of Wales, Cardiff, UK; ^2^School of Medicine, Cardiff University, Cardiff, UK

## Abstract

Yellow nail syndrome is a rare acquired condition of unknown aetiology associated with distinct nail discolouration/xanthonychia, pulmonary manifestations, and lymphoedema. Pleural plaques and diffuse pleural thickening are typically, although not exclusively, recognised as markers of prior commercial asbestos exposure. The presence of such biomarkers may assist an asbestos personal injury evaluation. A postmortem examination performed on a 72-year-old man with known long-standing yellow nail syndrome identified pleural plaques and diffuse pleural thickening. An evaluation of the occupational history identified no known asbestos exposure. Electron microscopic mineral fibre analysis detected no asbestos fibres. To the best of our knowledge, this is the only case of yellow nail syndrome in which these benign pleural changes are reported* ex asbestos*. Alternate causes for such pleural pathology were absent. There is merit in physicians and pathologists having an awareness of these new manifestations when considering claimed asbestos related changes during life and at postmortem.

## 1. Introduction

This article describes the new manifestations of bilateral parietal pleural plaques and diffuse visceral pleural thickening/fibrosis in a subject with yellow nail syndrome with no discernible asbestos exposure. Careful patient evaluation revealed no known occupational history and no asbestos bodies or asbestos fibres were detected by light microscopy or transmission electron microscopy, respectively. We describe the patient's ante-mortem history, post-mortem findings including histology, together with fibrous and non-fibrous mineral analysis. The extent of the pleural changes in this case was such to mimic those seen in asbestos related disease. In the setting of potential asbestos related personal injury claims and in the investigation of asbestos related deaths at post mortem, there should be an awareness that the spectrum of manifestations present in yellow nail syndrome may include pleural plaques and diffuse pleural fibrosis.

## 2. Case Presentation

A 62 year-old male was diagnosed with yellow nail syndrome in 2000. He had a long history of recurrent sinusitis from 1983 and had developed numerous respiratory tract infections since 1996. Primary lower limb lymphoedema was diagnosed in 2000. Shortly thereafter he developed recurrent, initially right pleural effusions. Repeat thoracocenteses had revealed cloudy, thick fluid, exudative in nature. A right video-assisted thoracoscopic pleural biopsy was performed which showed chronic inflammation and reactive mesothelial changes but no malignancy. The diagnosis of yellow nail syndrome was made when xanthonychia developed in 2000. The clinical course continued with the development of bronchiectasis in 2003, complicated by recurrent chest infections and bilateral effusions.

The subject had a history of heavy prior tobacco smoking and had worked as a general manager, policeman, clothing design director, and dark room technician.

He died in 2010 following an infective exacerbation of his bronchiectasis.

**Figure 1:**
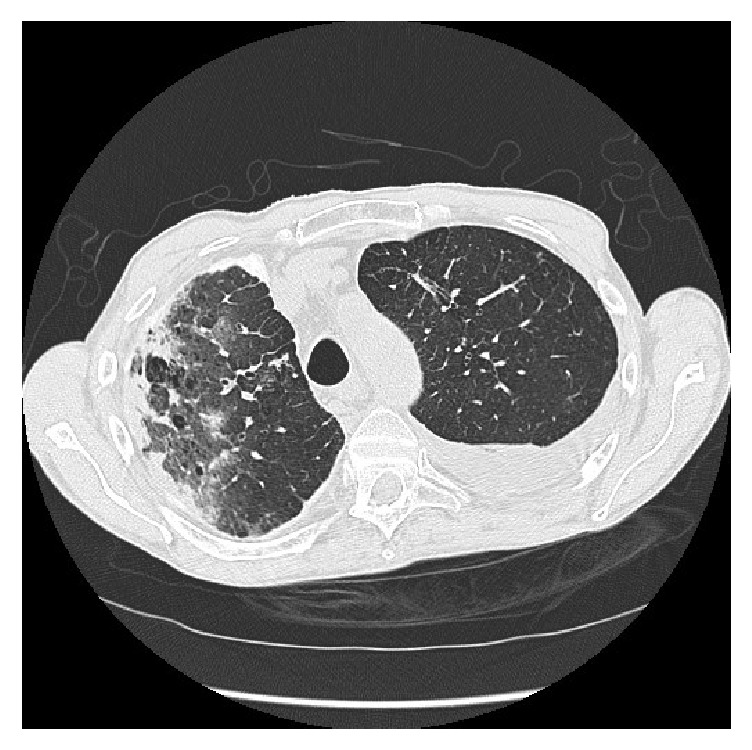
CT scan of chest, showing right airspace shadowing, bronchiectasis and diffuse pleural thickening.

A CT scan performed shortly before his death showed extensive right pleuroparenchymal disease, including right diffuse pleural thickening, bronchiectasis and right airspace shadowing (Figure 1).

**Figure 2:**
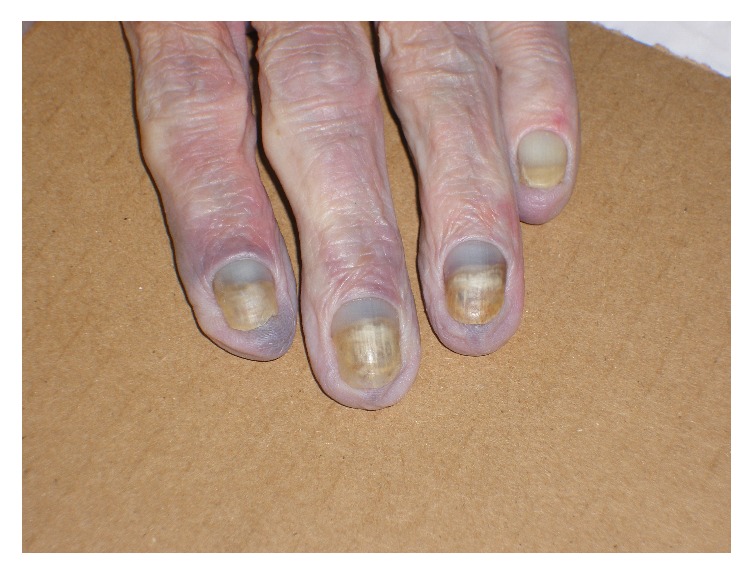
Yellow discolouration of the fingernails.

**Figure 3:**
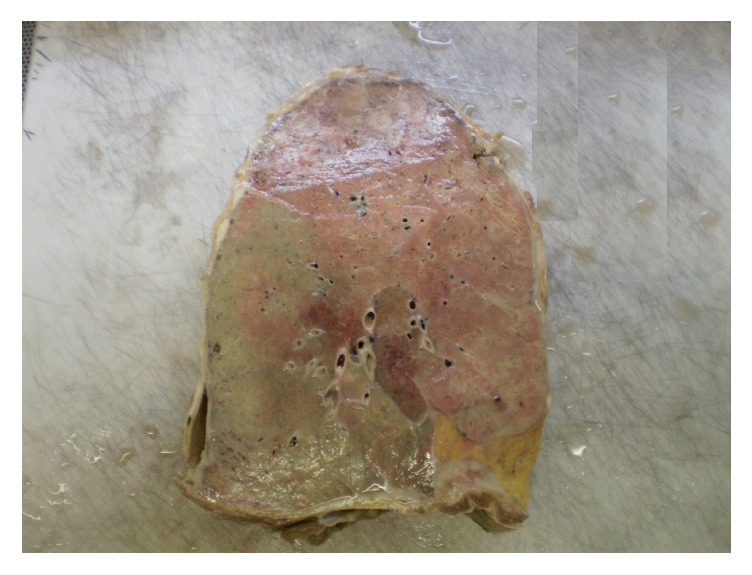
Diffuse pleural thickening affecting the entire visceral pleura.

**Figure 4:**
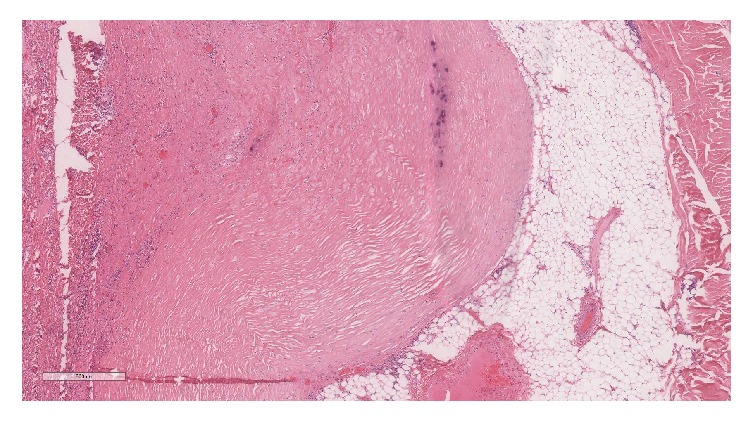
Basket-weave pattern of paucicellular collagen fibres in diffuse pleural fibrosis.

**Figure 5:**
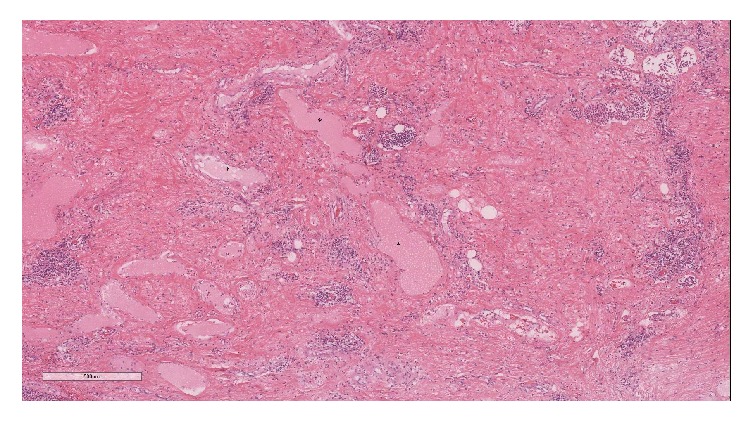
Dilated septal lymphatics (asterisk in lumen).

**Figure 6:**
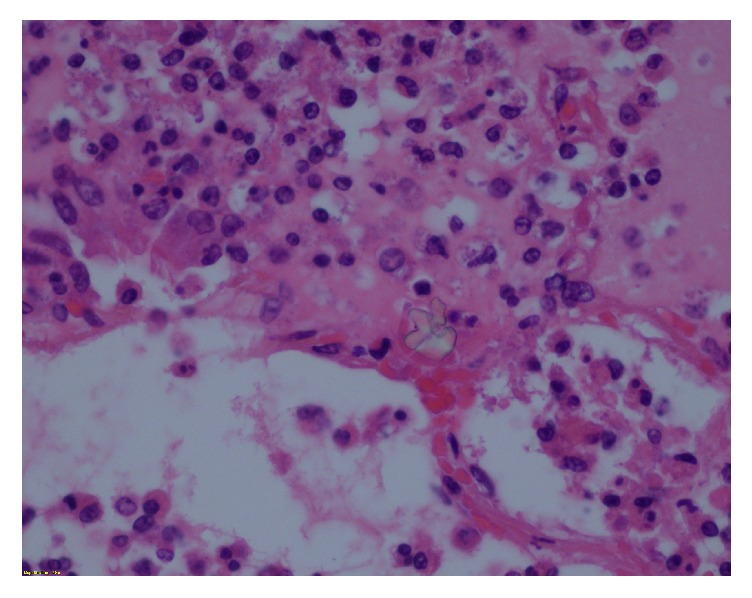
Platy form talc crystal in contralateral lung parenchyma.

A postmortem examination was performed. External examination revealed the presence of yellow discolouration affecting the finger- and toenails (Figure 2), along with bilateral lower leg oedema. Examination of the respiratory system showed extensive bilateral pleural adhesions, diffuse visceral pleural thickening and parietal pleural plaques (Figure 3). Microscopical examination confirmed the presence of paucicellular hyaline collagenous plaques with ‘basket-weave' pattern, bilateral diffuse pleural fibrosis composed of similarly paucicellular collagen, and occasional lymphoid aggregates (Figure 4). Septal lymphatics were noted to be markedly dilated (Figure 5). A right-sided lobar pneumonia with organisation was present. Careful inspection of multiple lung sections by light microscopy failed to detect any asbestos body formation. There was a talc pleurodesis reaction in the right pleural space. Within the lung tissue bilaterally, remote from pleura, platy form polarisable material was seen consistent with talc particulates (Figure 6). Other organ systems showed no significant abnormality.

Mineral analysis performed on tissue from the left lung by transmission electron microscopy and energy dispersive X-ray spectrometry detected: no commercial or noncommercial amphibole asbestos fibres or serpentine chrysotile asbestos. Fibrous mullite, a nonasbestos aluminium silicate, fibrous silica and muscovite were detected, confirming a technically successful analysis. Non-fibrous mineral analysis detected silicon, talc, aluminium, titanium, and iron.

Cause of death was recorded as yellow nail syndrome complicated by infection (lobar pneumonia) and diffuse pleural fibrosis (immediate cause).

## 3. Discussion

Yellow nail syndrome is a rare medical condition associated with yellow discolouration of the nails, lower limb lymphoedema and respiratory tract symptoms, including bronchiectasis and pleural effusions. The first series of patients were reported in 1964, by Samman & White, and since then less than 400 cases have been reported in the literature [1, 2].

The diagnosis of the syndrome requires at least 2 of the 3 features to be present simultaneously.

The aetiology of the disease is largely unknown, lymphatic impairment, autoimmune disorders, paraneoplastic manifestations, and exposure to titanium from implants and medications have been postulated to play a role in the development of the syndrome [2, 3].

Respiratory involvement was reported in 56-71% of the patients, with changes including pleural effusion, bronchiectasis, recurrent pneumonias, bilateral apical fibrosis, patchy alveolar infiltrates, and cystic lesions [2].

Histopathological examination of the pleura either is noncontributive or shows features of chronic fibrosing pleuritis [4].

We report a case of yellow nail syndrome in which the extent of pleural pathology was so marked as to mimic asbestos-related disease in a subject with no proven asbestos exposure.

Diffuse visceral pleural thickening/fibrosis and parietal pleural plaques are an exceptional finding in yellow nail syndrome.

Diffuse pleural thickening is defined as a uni- or bilateral thickening of the visceral pleura, at least 5 mm in thickness and involving at least one third of the affected lung surface.

Histologically it is characterised by a basket-weave pattern of pleural fibrosis with subpleural parenchymal interstitial fibrosis superficially extending into the underlying lung [5].

While diffuse pleural thickening is commonly associated with exposure to commercial forms of asbestos, it can also be caused by collagen vascular diseases (systemic lupus erythematosus, rheumatoid arthritis), drugs (methysergide and chemotherapy), infection (TB or chronic fungal), and sarcoidosis and can also be idiopathic in nature.

Pleural plaques are well-circumscribed lesions of the parietal pleura, composed of hyalinised, acellular collagen with a basket-weave pattern. Their presence is commonly associated with prior exposure to commercial forms of asbestos; however they can also be caused by local trauma and focal inflammation or even appear idiopathic in nature.

In most cases of pleural thickening and pleural plaques, the clinician and pathologist will consider asbestos-related disease; however this paper highlights that in the right clinical context, yellow nail syndrome can also mimic these changes and must be ruled out.

Mineral fibre analysis has an important role in determining an individual's inhaled, deposited and retained mineral content. Transmission electron microscopy and energy dispersive X-ray spectrometry was performed in this case and identified no asbestos fibres. A variety of nonfibrous minerals were identified including various metals and talc. The role of titanium in yellow nail syndrome has been suggested. To this end, it is of note that metal analysis detected low level titanium although the significance of this in isolation is uncertain. Talc was histologically observed and mineralogically identified in contralateral lung to the site of talc pleurodesis, supporting the notion that talc instillation may circulate remote from the pleural space. It is important for the clinicians to be aware that yellow nail syndrome may induce diffuse pleural fibrosis and pleural plaques without a history of asbestos exposure. It is also of equal importance that the pathologist performing the postmortem examination be aware of these new manifestations of yellow nail syndrome when investigating potential asbestos related deaths.
